# SARS-CoV-2 versus other minor viral infection on kidney injury in asymptomatic and mildly symptomatic patients

**DOI:** 10.1080/21505594.2022.2107602

**Published:** 2022-08-04

**Authors:** Ya-Chieh Chang, Ding-Jie Lee, Chia-Ling Helen Wei, Chung-Han Pa, Chien-Chou Chen, Hsi-Chih Chen, Yu-Tien Chang, Han-En Wang, Pauling Chu, Kuo‐cheng Lu, Chia-Chao Wu

**Affiliations:** a Department of Internal Medicine, Division of Nephrology, Tri-Service General Hospital, National Defense Medical Center, Taipei, Taiwan; b Department of Internal Medicine, Division of Nephrology, Taoyuan Armed Forces General Hospital, Taoyuan, Taiwan; c School of Public Health, National Defense Medical Center, Taipei, Taiwan; d Department of Medicine, Division of Nephrology, Taipei Tzu Chi Hospital, Buddhist Tzu Chi Medical Foundation, New Taipei City, Taiwan; e Department and Graduate Institute of Microbiology and Immunology, National Defense Medical Center, Taipei, Taiwan

**Keywords:** SARS-CoV-2, respiratory viruses, influenza virus, kidney damage, asymptomatic

## Abstract

Coronavirus disease 2019 (COVID-19) is caused by severe acute respiratory syndrome coronavirus 2 (SARS-CoV-2) and has become a global pandemic since December 2019. Most of the patients are mild or asymptomatic and recovered well as those suffered from other respiratory viruses. SARS-CoV-2 infection is supposed to demonstrate more sequelae. Acute kidney injury (AKI) is common among COVID-19 patients and is associated with disease severity and outcomes. Only a few studies focused on a detailed analysis of kidney damage in asymptomatic or mildly symptomatic COVID-19 patients. Whether any minor viral infection is likely to exhibit similar minor effect on renal function as COVID-19 is still unclear, and the definite pathophysiology of viral invasion is not fully understood. Currently, the proposed mechanisms of AKI include direct effects of virus on kidney, dysregulated immune response, or as a result of multi-organs failure have been proposed. This study will discuss the difference between COVID-19 and other viruses, focusing on proposed mechanisms, biomarkers and whether it matters with clinical significance.

## Introduction

Coronavirus disease 2019 (COVID-19) is caused by severe acute respiratory syndrome coronavirus 2 (SARS-CoV-2), which escalated into a global pandemic since December 2019. It already resulted in more than 500 million cases and more than 6 million deaths globally. Initially, it mainly affects the lungs and is considered a unique respiratory virus-induced illness, but now multiple organ systems are recognised to be affected. According to current evidence, extrapulmonary involvement in COVID-19 patients has been reported, and the affected organs and systems include myocardium, gastrointestinal tract, vascular endothelium, neurological system, and kidneys [1].

Acute kidney injury (AKI) is the most frequently encountered extrapulmonary manifestation among COVID-19 patients, and the reported rate are extremely variable (1%–80%), which is considered to be associated with disease severity and outcomes [2,3]. Previous systemic review showed that the pooled incidence of AKI in COVID-19 patients was 19.45%, which seems to be relatively higher than other respiratory coronavirus infections (5%–15%) [1,4]. Although the definite pathophysiology of AKI is still in the exploration stage, several studies discriminated the possible mechanisms. Further kidney damage might be due to direct effects of virus on kidney, dysregulated immune response, or as a result of multi-organs failure [3]. Clinical signs suspicious of COVID-19 include cough, dyspnea, sore throat, fever, malaise, olfactory loss, and myalgia. In fact, no obvious difference from other respiratory virus infection, such as influenza virus and coronavirus, caused the SARS pandemic of 2003 [5–7].

Influenza A is the most common infectious disease compared with COVID-19 because of similar transmission routes, clinical manifestation, and complications. AKI had also been reported in influenza A patients, but it usually occurred in those severe illness group [8]. Asymptomatic COVID-19 patients or those with mild illness account for the majority, especially individuals infected by current Omicron variant. Only a few studies exist focused on detailed analysis of kidney damage on COVID-19 patients with mild illness. Whether any minor viral infection is likely to exhibit a similar minor effect on glomerular filtration rate (GFR) reduction like SARS-CoV-2 infection is still unclear. These proposed mechanisms and whether it matters with clinical significance are also unclear. This study aimed to discuss the unique feature of SARS-CoV-2 that differ considerably from other minor viral infection on kidney injury in mildly symptomatic and asymptomatic patients.

### Point 1. Whether any minor viral infection is likely to demonstrate similar minor effect on kidney injury as SARS- CoV-2 in asymptomatic or mildly symptomatic patients?

First, the SARS-CoV-2 virus is a unique and novel virus, which belong to Betacoronavirus and is highly transmissible in humans. The positive-sense single-stranded RNA is contained within envelope and has approximately 80% genetic similarity with SARS-CoV and is less similar to the nucleotide sequence of Middle East respiratory syndrome coronavirus (about 50%) [9]. The SARS-CoV-2 structure contains four structural proteins including spike (S), envelope (E), membrane (M), and nucleocapsid (N) proteins. The S protein of SARS-CoV-2 has intense binding affinity to angiotensin-converting enzyme 2 (ACE2) receptors on host cell surface and their interaction initiates the viral cell entry. Furthermore, cleavage of the S protein activates the infection process. Binding through cathepsin L or transmembrane serine protease 2 (TMPRSS2) can promote membrane fusion, then viral RNA is released into infected cell [10–12]. To current consensus, ACE2 and TMPRSS2 are regarded as the crucial mediators for the entry step of SARS-CoV-2, so tissues enriched with these two proteins automatically become candidate for viral infection. That is why COVID-19 patients present more broadly extrapulmonary manifestations. Kidney is also supposed to be the target of SARS-CoV-2, and direct infection can lead to tubule-interstitial fibrosis without any systemic effect had been proved through stem cell-derived kidney organoid models by Jitske Jansen et al [13].

**Figure 1.**
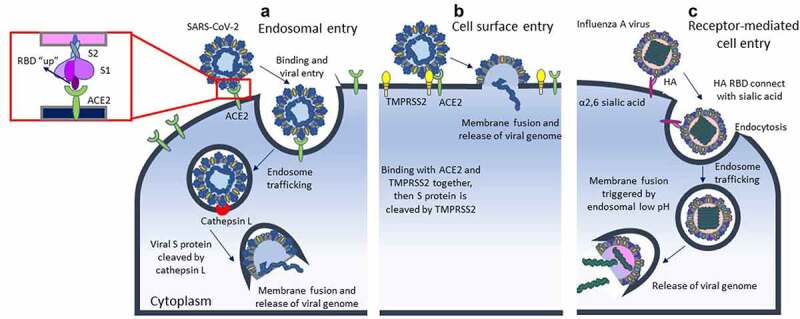
Difference of viral cell entry between SARS-CoV-2 and influenza a virus.

Except for SARS-CoV-2, other sarbecoviruses like SARS-CoV do not exist in the step of S protein cleaved by furin-like proteases during viral maturation [10]. As for further respiratory virus, influenza A viruses (IAVs) may affect host cell via glycan-dependent entry. IAVs initiate infection process by using the haemagglutinin (HA) RBD to connect to sialylated glycoconjugates on infected cell, and then, endocytosis is triggered. (Figure 1). The participant of ACE2 and TMPRSS2 are not necessary for IAVs cell entry; nevertheless, TMPRSS2 still play a role in proteolytic activation of some H1N1 subtype IAVs. It demonstrates no involvement in viral maturation of incoming virions but cleavage nascent HA within the host cell [14]. Prior studies found that IAVs infection may also increase ACE2 expression on airway epithelial cell, which might possibly explain the high rates of co-infection of COVID-19 and influenza [15]. Despite these viruses exhibit close association, they still show obvious difference on pathophysiology and disease characteristics. Selected comparisons between SARS-CoV-2, SARS-CoV, and influenza virus are summarised in Table 1.

The SARS-CoV-2 invade host cell via two pathways, the first form is endosomal entry (A). The virus uses the receptor binding domain (RBD) on S1 to bind to ACE2 with S1 “up” conformation, then internalized into infected cell. During endosome trafficking, viral S protein is cleaved at the S2ʹ site by cathepsin L, a furin-like proprotein convertase, and then uncoated viral RNA can be released into host cell. The second form is cell surface entry (B), SARS-CoV-2 bind with TMPRSS2 and ACE2 together, then TMPRSS2 activates the S protein for membrane fusion. Different to SARS-CoV-2, influenza A virus does not need the assistance of ACE2 or TMPRSS2 for cell entry. It infect host cell through receptor-mediated cell entry (C), which use RBD of HA on viral surface to connect with α2,6 sialic acid. Endocytosis occur and the following membrane fusion will be triggered in the acidic environment of endosomes.

**Table 1.** Comparison between different respiratory viruses.

Parameters	SARS-CoV-2	SARS-CoV	Influenza
Structure	Single-stranded, positive-polarity RNA	Single-stranded, positive-polarity RNA	Single-stranded, negative-polarity RNA
Receptor	ACE2	ACE2	Sialic acid-containing molecules
Priming process [16]	Proteolytic cleavage by TMPRSS2, cathepsin L and furin	Proteolytic cleavage by cathepsin L and furin	HA processing by trypsin-like proteases
Transmission pathway [17]	Droplet, airborne, contact	Droplet, airborne, contact	Droplet, airborne, contact
Incubation period [18]	2–14 days	2–14 days	2 days
Tissues involved [18]	Respiratory system, heart, kidney, intestine, nervous system	Respiratory system, immune cells, gastrointestinal tract, kidney, brain	Upper and lower respiratory tract
Association of viral load [19,20]	No absolute correlation	No absolute correlation	Correlated positively with disease severity
Sequelae [21,22]	Pulmonary fibrosis, lung function impairment, thromboembolic events, myocardial fibrosis, arrhythmias, renal function decline, prolonged viral fecal shedding, neuropsychiatric disorder	Pulmonary fibrosis, lung function impairment, muscle weakness, neurological and psychobehavioural disorder	Susceptibility to secondary infection, pulmonary fibrosis

SARS-CoV-2 : severe acute respiratory syndrome coronavirus 2.

ACE2 : angiotensin-converting enzyme 2.

TMPRSS2 : transmembrane serine protease 2.

HA : haemagglutinin.

Second, not all the mechanisms of virus-induced kidney damage are the same. Divergent pathways of renal injury exist by viral infections, including direct invasion leading to cytopathic injury, inflammation-mediated tissue destruction and alterations in host haemodynamic responses [23]. Nearly, all the virus infection may induce systemic immune response, but not all of the virus can invade the kidney directly. As mentioned above, SARS-CoV-2 tend to attack organs rich in ACE2 and TMPRSS2 because of their necessity for infection process of COVID-19. An organotropism is found beyond the respiratory tract, including the kidneys, liver, heart, and brain. According to both the protein expression profile and mRNA-sequencing data, we can find highly expression of ACE2 and TMPRSS2 across multiple cell types in the kidney. The most abundant site is epithelial cell on renal tubule [24], and other nephron subunits include endothelial cells and mesangial cells. However, it is interesting to note that the main distribution of these two key proteins is distinct. ACE2 is enriched in proximal tubule and podocyte, whereas TMPRSS2 is mostly located in distal tubule and intercalated cells. Although double positive cells are rarely detected in kidney, evidences of direct invasion to above regions had been reported [25]. In view of this, we suppose that SARS-CoV-2 enters proximal tubules assisted by ACE2 and cathepsin L. Another potential protein may cooperate with TMPRSS2 to facilitate direct invasion to distal tubule via non-ACE2 pathway. Additionally, some studies also propose the theory that transmembrane glycoprotein CD147 among with cyclophilin A serve as an alternative receptor for SARS-CoV-2 entry, which is present on tubular epithelia and podocyte [26].

In spite of sialic acid α 2,6-galactose-linked receptors expression on renal glomeruli of pig [27], its distribution in human still confines to ileal epithelium, ciliated and non-ciliated cells of the respiratory tract [28]. No sufficient evidence showing kidney damage from influenza infection is attributable to direct viral invasion. In contrast to IAVs, SARS-CoV-2 exists diverse entry pathway to impact the kidney. Therefore, renal tissue is supposed to have higher susceptibility to SARS-CoV-2 infection and may be a reasonable explanation that AKI is prevalent among COVID-19 patients [29,30]. The degree of virus-induced inflammatory status or tissue invasion is heterogeneously different. Initially, viraemia occur first and then originate systemic inflammation or changed haemodynamic response subsequently induced kidney damage. Then, the virus can enter kidney cells directly or followed by penetration of the glomerular barrier into renal tubules. As stated above, we can understand that SARS‐CoV‐2 is quite unique and different from other viral infections structurally, which demonstrates the ability to induce kidney damage via assorted mechanisms.

### Point 2. Whether evidence exists to support the pathogenesis of SARS- CoV-2 on the kidney in (asymptomatic or mildly symptomatic) COVID-19 patients?

First, renal tropism of SARS-CoV-2 and evidence of directly infected renal parenchyma had been reported. SARS-CoV-2 exhibits an extrapulmonary organotropism and tends to infect ACE2-enriched tissues, the kidney among the rest. SARS-CoV-2 binds to ACE2 as a receptor for cellular entry. The ACE2 RNA was expressed nearly 100-fold more in the kidney than in the lungs. ACE2 and TMPRSS, crucial roles for viral entry, were highly expressed in the brush border of tubular cells but less in podocytes [31]. Thus, the absence of predominant glomerular involvement may be resulting from the ACE2 distribution difference.

The most common histopathologic features of autopsied COVID-19 kidney specimens include tubulointerstitial nephritis, acute tubular necrosis, and lymphocyte infiltration in varying degrees. Also, viral infection associated-syncytia were observed. Otherwise, both nucleocapsid protein antigen and viral RNA of SARS-CoV-2 have been detected in the kidney tissues by immunostaining and molecular biotechnology. Furthermore, recognition of coronavirus-like particle structure in podocytes and proximal renal tubules by electron microscopy and live virus recovered from kidney tissue have also been proved [32]. Recently, Huang et al. disclosed the separation and identification of virus particles from urine sample of affected patients. These virions are considered to be kidney-originated and may pass through glomerulus, then enter the urinary stream directly [33]. The viral load in urine sediments detected by quantitative real time polymerase chain reaction (qRT-PCR) also display a positive correlation with incidence of AKI and mortality [34]. All the evidence supports that SARS-CoV-2 might exhibit viral tropism and direct persistence, which is a potential explanation of commonly occurring kidney injury in patients with COVID-19 [24].

Second, the pathophysiology and mechanisms of kidney damage in COVID-19 patients have not been fully elucidated. Observation on the existance of coronavirus in the kidneys and urine supports the hypothesis that virus can directly invade the kidneys. However, some people claim that the presence of viral protein may not equal to direct viral damage, and these findings might not have absolute specificity [32]. A retrospective cohort study showed that significant associations of SARS-CoV-2 viral load are found with in-hospital AKI, which may indicate the direct effect of SARS-CoV-2 virus on the kidney [35]. In a Belgium study, Werion et al. present a proximal tubular dysfunction in patients with COVID-19 [36]. A high prevalence of hypokalaemia was found among infected patients because of continuous renal potassium loss resulting from the presence of disordered rennin-angiotensin system activity [37]. These data replenish current evidence regarding SARS-CoV-2 presence and potential infection in the kidney. Nevertheless, some studies suppose that relatively low viral load with uneven distribution detected in the renal tissues may not possess the ability to attack kidneys extensively [38].

Similar to nearly all, the virus can induce systemic inflammatory response, strong presence of CD68+ macrophages and membrane attack complex depositions in the tubulointerstitium noted in the kidney of COVID-19 patients, which can further accelerate and amplify renal injury. As discussed previously, the virus may also invade renal parenchyma and cause further renal complications like acute tubular necrosis. Although podocytes are not predominantly affected site, collapsing focal segmental glomerulosclerosis due to direct viral effect has also been reported. The above kidney damage may also occur through dysregulation of the immune responses and microthrombi formation and the aggregation of fibrin deposition, which leads to acute ischaemia and AKI resulted from systemic inflammation [32].

AKI may also occur in patients infected by other respiratory viruses, especially influenza. Prior studies showed approximately one third of hospitalised patients with pandemic influenza A (H1N1) virus infection presented with AKI [39]. Like COVID-19, the definite pathogenic mechanisms that develop AKI remain unclear. Several hypotheses had been proposed, including severe infection-related rhabdomyolysis, acute immune complex-mediated glomerulonephritis, acute tubular necrosis due to renal hypoperfusion, or disseminated intravascular coagulation [8]. Although one case series displays that IAV can be found in the cytoplasma of glomerular macrophages in four patients, no sufficient evidence of direct kidney invasion exists [40].

**Figure 2.**
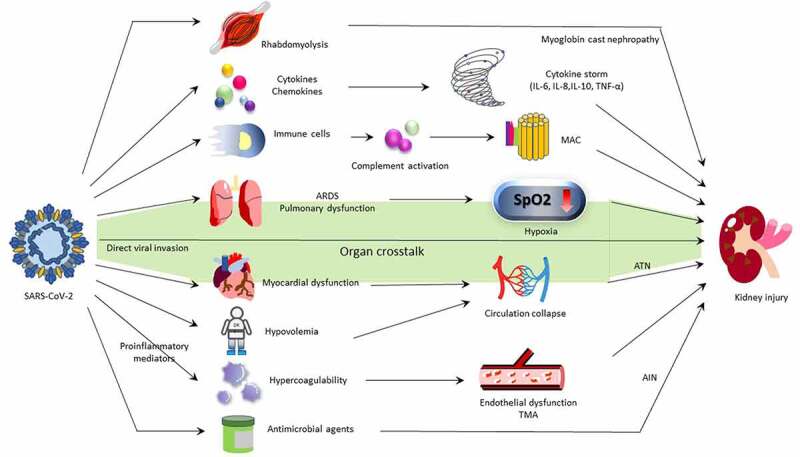
Mechanism of COVID-19-associated AKI.

The proposed mechanisms of COVID-19-induced kidney damage are demonstrated in Figure 2. The COVID-19-induced kidney damages are involved through both direct and indirect mechanisms. The consensus report of the 25^th^ Acute Disease Quality Initiative Workgroup concerning COVID-19-associated AKI stated that the pathophysiology and mechanisms of AKI in infected individuals still exist uncertainly and seem to be multifactorial. Also, they stated that whether kidney damage is caused by systemic inflammation and immune dysfunction remains controversial.

AKI in COVID-19 patients is supposed to be multifocal; it may be caused by immune system dysregulation or complement activation, similar to other respiratory virus infection. Rhabdomyolysis occurs in several virus infection with severe illness, and the obstruction of renal tubules by myoglobin casts can also induce AKI. Proinflammatory mediators enhance blood coagulation and result in endothelial dysfunction or even thrombotic microangiopathy (TMA). Erythrocyte aggregates may induce obstruction of the capillary lumen and ischemic injury in the kidneys. Severe COVID-19 patients usually receive broad-spectrum antibiotics during acute-phase infection, so drug-induced acute interstitial nephritis should also be considered. SARS-CoV-2 infection can cause both pulmonary and myocardial dysfunction, and AKI may occur through these pathways of organ crosstalk. Finally, SARS-CoV-2 is found to have direct viral invasion to kidney, a unique pathophysiology of renal damage. There is no evidence that other respiratory virus has the same ability.

Third, further work is needed to clarify the precise aetiology of COVID-19-associated AKI. Although renal tropism is supported by majority of current researches and some studies even show evidences of direct viral invasion to the kidney, acute tubular injury is reported as the main histologic lesion [38]. This finding implies the leading cause of kidney damage might be resorted to systemic dysfunction, such as acute respiratory distress syndrome, hypotension, nephrotoxin exposure or severe inflammatory response. Even though direct kidney infection of SARS-CoV-2 is indeed, it is considered to play a relatively minor role. For the current data, AKI caused by either a direct cytotoxicity of SARS-CoV-2 or by indirect immune-mediated pathogenesis is all possible. Further investigation about the definite pathophysiology and the effects of COVID-19 infection on kidney is our first imperative.

### Point 3. Whether slight difference in GFR in asymptomatic COVID-19 patients indicated any clinical significance and value?

First, no evidence exists to support all viral infection in asymptomatic patient will induce similar minor effect on GFR as COVID-19. Whenever there is overt symptoms, viral infection may act on kidneys through the “direct effects” of invasive disease and/or “indirect effects” of inflammatory responses on kidneys. The virus-induced systemic inflammatory responses may exhibit effects on GFR; however, the degree is diversified. One retrospective cohort study investigated the renal presentations of COVID-19 victims with different disease severity. Proteinuria and microscopic haematuria tend to occur among severe individuals, and both of these manifestations are independent risk factors for mortality [41]. No evidence was provided, and little is known about the effects on GFR among asymptomatic patients. Take an example of IAV infection. Despite the fact that development of IAV-related kidney injury has not been depicted, heterogeneous and dynamic prevalence of asymptomatic influenza virus infections was founded [24,42].

Only a small amount of studies focusing on the viral infections were involved in kidney allograft function [43]. A variety of pathogenic viruses have been proven to cause renal complications in kidney transplant patients. Either acute or chronic graft dysfunction can be resulting from adenovirus and polyomavirus BK infection. Besides, adenovirus can also bring out haemorrhagic cystitis and tubulointerstitial nephritis. These viral infections may demonstrate effects on the long-term allograft survival [43,44]. As for the impact of SARS-CoV-2 infection in renal transplant recipient, most patients present cough, myalgia, chills, and fatigue, similar to general individuals. However, hospitalization is usually required and increased overall mortality has also been reported [45]. Otherwise, renal function decline may also occur during initial infection, regardless of the clinical presentation or age [46]. Hence, findings of COVID-19 in asymptomatic and mild patients are unique and informative.

Second, those asymptomatic patients are not absolutely caused by minor viral infection. Although some studies, mostly published in 2020, supported that a higher SARS-CoV-2 viral load might be associated with growing disease intensity and mortality [47–49]. With mounting cases accumulated rapidly and further exploration of COVID-19, the significant correlation between initial viral load and disease progression seems to be overthrown [50,51]. Because of different host immune response, viral load is dynamic and vary over the course. Prior study has tried to constitute a model framework of variation of viral load for precise treatment policy, but additional investigation is needed [52]. Besides, definite immune pathway when initial exposure to SARS-CoV-2 and the crucial determinant of symptomatic presentation remain unknown [53]. Asymptomatic COVID-19 patients with higher viral load have also been broadly reported. This phenomenon represents that asymptomatic are not equal to undetectable or untransmittable, on the country, it might be the potential threat from the iceberg [54–56]. Hence, SARS-CoV-2 viral loads may aid in the risk stratification of patients with COVID19 but not always demonstrate a positive correlation with severity of clinical manifestation. Future research should focus on SARS-CoV-2 viral load dynamics and its role in disease pathogenesis.

Even if patients did not present respiratory symptoms or recovered from COVID-19 clinically, viruses may still remain hidden in our body and attack extrapulmonary organs consequently, especially in groups with immunodeficiency. The concept of extrapulmonary reservoir indicates that there exists other transmission pathway rather than respiratory secretions, regardless of the detected viral load. Otherwise, SARS-CoV-2 is supposed to increase the risk of organ dysfunction [57].

Third, is slight difference in GFR in asymptomatic COVID-19 patients meaningful? The exact dynamic changes of renal functions in those asymptomatic ones still need further explorations. According to previous studies, there is a positive correlation between COVID-19-associated renal complications and mortality rate. AKI is considered to be independent risk factors for both long-term renal outcome and all-cause in-hospital death in infected patients [58].

Different to influenza, which may develop sequelae limited to respiratory system, such as susceptibility to secondary bacterial infection and pulmonary fibrotic changes [59], Benjamin Bowe et al. demonstrated that COVID-19 may experience post-acute sequelae involving pulmonary and broad extrapulmonary organ system. Otherwise, the study exhibited that COVID-19 survivors demonstrate an increased incidence of AKI, renal function decline, end stage kidney disease and major adverse kidney events compared with those non-infected controls. Although a positive relation exists between eGFR loss and disease severity, increased risk of renal sequelae mentioned above is still evident among those individuals with mild symptoms or even asymptomatic ones. Based on this statistically significant finding, specialists strongly suggested to establish post-acute COVID-19 clinics because majority of COVID-19 victims are non-hospitalised, who may neglect further development of sequelae easily [60]. The international HOPE COVID‑19 (Health Outcome Predictive Evaluation for COVID 19) Registry regards eGFR at admission as an independent factor of prognosis. When eGFR is below 150 mL/min/1.732 m^2^, including patients already have chronic kidney disease, a tendency towards conflicting relationship between renal function and all-cause mortality is found [61]. Hence, we can tell that slight difference in GFR in asymptomatic COVID-19 patients is surely meaningful.

Finally, biomarkers are linked with changes of renal function in asymptomatic and mildly symptomatic COVID-19 patients. Some asymptomatic carriers also developed deterioration of renal function in various degrees during hospitalisation. Although it is difficult to accomplish in the real world, early detection through contact tracing may efficiently avoid presymptomatic transmission and reduces the occurrence of critical cases [62]. Not all one is really assessing kidneys in asymptomatic people, so we just do not know what is going on. Some novel biomarkers like cystatin C, neutrophil gelatinase-associated lipocalin (NGAL), and kidney injury molecule 1 (KIM-1) are more specific for early detection of AKI. Recently, coronavirus detection in urine sediment suggests further kidney invasion and is associated with risk of AKI and poor prognosis. Considering this urinary viral detection is expensive and not easily accessible, it is also not practicable to utilise, not to mention that performing renal biopsy in such asymptomatic individuals. The majority of the studies demonstrated positive correlations between the use of CRP and neutrophil-to-lymphocyte ratio (NLR) in COVID-19 with disease severity or mortality. Elevation in neutrophil counts, CRP, and NLR had also been provided as useful biomarkers to link with the worsening of the renal function in asymptomatic patients. These biomarkers are non-invasive, convenient, and easy practice for clinical application [47]. Long-term outcome of renal function progression in asymptomatic COVID-19 patient still need further evaluation.

## Conclusion

SARS-CoV-2, different from other familiar respiratory viruses, had destroyed numerous families and forced the world to advance amidst turbulence. Through abundant researches focused on COVID-19, we understand that SARS-CoV-2 enter infected cell mainly mediated by ACE2 and TMPRSS2. Nevertheless, more potential pathways for renal invasion exist and the kidney is supposed to have strong susceptibility. Although there is still controversy, several studies had proved the theory of direct kidney infection and consequently tubulointerstitial injury. Besides renal tropism, systemic inflammatory response and organ crosstalk also participate in the development of AKI and eventually impact disease outcome. Even if patients with mild illness or those asymptomatic individuals may still exhibit high viral load. Condition of viral replication and influences on the human body are present as a dynamic variation. Most of the COVID-19 patients do not have life-threatening manifestations during early infection but a quantity of them do develop extrapulmonary sequelae at post-acute phase, which indicated the importance of regular health survey. Due to frequent mutation of this troublesome coronavirus, many issues remain unclear and need further exploration. We all hope improvement of technology and knowledge can terminate the disaster as soon as possible.
